# People’s Willingness to Pay for Dental Checkups and the Associated Individual Characteristics: A Nationwide Web-Based Survey among Japanese Adults

**DOI:** 10.3390/ijerph20054145

**Published:** 2023-02-25

**Authors:** Katsuo Oshima

**Affiliations:** Department of Dental Technology, The Nippon Dental University College, Tokyo 102-8159, Japan; oshima@tky.ndu.ac.jp; Tel.: +81-3-3265-8815

**Keywords:** dental checkups, willingness to pay, oral health, web-based survey, Japan, health policy, socioeconomic factors, economics

## Abstract

This study aimed to determine the willingness-to-pay (WTP) values for dental checkups and analyze the association between the values and individual characteristics. This cross-sectional study was conducted using a nationwide web-based survey, and 3336 participants were allocated into groups that received regular dental checkups (RDC; *n* = 1785) and those who did not (non-RDC; *n* = 1551). There was a statistically significant difference in the WTP value for dental checkups between the RDC (median: 3000 yen [22.51 USD]) and non-RDC groups (2000 yen [15.01 USD]). In the RDC group, age 50–59 years, household income <2 million yen, homemaker and part-time worker employment status, and having children were significantly associated with decreased WTP values; male sex, household incomes ≥8 million yen, and tooth brushing ≥3 times daily were associated with increased WTP values. In the non-RDC group, age ≥30 years, household incomes <4 million yen, and having ≥28 teeth were significantly associated with decreased WTP values; household income ≥8 million yen was associated with increased WTP values. Conclusively, WTP values for dental checkups were lower in the non-RDC group than in the RDC group; in the non-RDC group, those with lower household income aged ≥30 years were more likely to propose lower WTP values, suggesting the need for policy intervention to improve access to RDC.

## 1. Introduction

Maintaining good oral health hygiene entails routine dental checkups and consulting dentists to diagnose underlying dental diseases. Apart from maintaining masticatory function, good oral health conditions contribute to an enhanced quality of life [1,2] and reduce the impact of systemic non-communicable diseases [3,4]. Therefore, creating health policy plans to promote the importance of regular dental checkups among the population is essential.

However, access to dental services such as routine checkups is affected by the health insurance system in each country. In countries with extensive dental insurance policies, the use of insurance coverage contributes to an increase in dental service utilization [5,6,7]. Moreover, studies in several countries have shown that an individual’s economic situation is one of the factors associated with access to dental services [8,9,10,11]. That is, even if they perceive the need for a specific dental service, their decision depends on their own income limitations and whether they are willing to spend resources for that dental healthcare service.

The contingent valuation method (CVM) is used for measuring the benefits of healthcare services. This method evaluates the Willingness to Pay (WTP), which is “the maximum amount of monetary value that an individual would be willing to sacrifice to obtain the benefit of that healthcare service,” through questionnaires or face-to-face interviews based on a hypothetical scenario regarding the healthcare service [12,13,14]. Several studies have reported on WTP for dental treatments, including preventive treatment of dental caries [15,16], periodontal disease [17], dental implant treatment [18,19], and orthodontic treatment [20]. The findings of studies on WTP can be used to perform economic evaluations of desired healthcare services by the general population and are expected to be a resource for policy planning regarding oral health [13]. As such, several dental WTP studies have demonstrated the distribution of WTP values to dental healthcare services based on the responses of survey participants and have shown that high/low WTP values are associated with individual characteristics such as socioeconomic status [13,15,16,17,18,20].

There have been few WTP studies on dental checkups [21,22]. A Finnish study [21] evaluated the WTP for dental checkups in 7-year-olds as one of the healthcare services targeted toward medical and dental students; however, the study did not target the general population and was limited to the evaluation of pediatric dental checkups. Further, a Japanese study [22] evaluated the WTP for dental checkups in regular visitors and infrequent visitors; however, this study only surveyed patients in dental clinics, and the findings were not applicable to the general population.

In Japan, a universal health insurance coverage system was established in 1961, and most dental treatments are covered by the medical insurance system, with patients paying a co-payment cost of 10–30% of the treatment cost [23]. However, since the medical insurance system covers only the treatment of diseases, services such as dental checkups, wherein the presence or diagnosis of oral diseases is not certain, are not covered by the insurance system [23]. The Ministry of Health, Labour, and Welfare of Japan released a report in 2022 on its oral health policy plan, which states that the most recently published rate of regular dental checkups among the population was 52.9%; therefore, it is necessary to continue to improve this rate in the future [24,25]. In 2022, the Japanese government released a basic policy of “universal oral health checks,” which allows all citizens to receive dental checkups throughout their lives [26]. Therefore, a study focused on understanding the WTP for receiving dental checkups to assess the economic value of targeting the general population on a nationwide scale, not just patients visiting dental clinics, would have policy implications. In particular, understanding the characteristics of individuals with low WTP values for dental checkups from among those who do not receive regular dental checkups will provide evidence for planning policies to further improve the rate of regular dental checkups; however, no such studies have been conducted.

The purpose of this study was to obtain and compare the WTP values for dental checkups in two groups of study participants (those who received regular dental checkups and those who did not) by using data from a nationwide web-based survey and to analyze the study participants’ individual characteristics associated with high/low WTP values for each of the two groups. Therefore, the null hypothesis for this study was set as follows: (1) there is no difference in the WTP values for dental checkups between those who did and did not receive regular dental checkups, and (2) there is no association between the WTP values and individual characteristics of each study group.

## 2. Materials and Methods

### 2.1. Study Design and Study Participants

This was a cross-sectional study conducted using a web-based survey in accordance with the STROBE statement. The study participants were recruited from among the registrants of a research company specializing in web-based surveys (Macromill, Inc.; Tokyo, Japan), which has approximately 1.3 million registered residents in Japan. The age criterion for the study participants was 20–69 years. Japan has a population of approximately 75 million residents aged 20–69 years. Based on an error margin of 2%, 95% confidence coefficient, and 50% population proportion, a minimum sample size of 2401 participants was required. Additionally, in a Ministry of Health, Labour and Welfare report, a web-based survey was used to assess the status of receiving dental checkups, which covered 3556 individuals aged ≥20 years on a nationwide scale [25,27]. Accordingly, the study targeted a sample size of 3200 participants aged 20–69 years. Finally, a total of 3336 participants were randomly selected from the research company’s database of registrants using a quota sampling method based on the Japanese national census population [28]. The distribution of the study participants was divided according to gender (men: 50.3%, women: 49.7%), age group (20–29 years: 15.7%, 30–39 years: 18.3%, 40–49 years: 23.8%, 50–59 years: 21.7%, and 60–69 years: 20.6%), and regional category (Hokkaido region: 4.2%, Tohoku region: 6.8%, Kanto region: 35.9%, Chubu region: 18.0%, Kinki region: 15.9%, Chugoku region: 5.4%, Shikoku region: 2.8%, and Kyushu region: 11.0%), which reflects the representation of the Japanese population [28].

As this study used a web-based survey, all study participants had to answer each question before they could proceed to the next question. Additionally, all participants completed the survey; thus, no missing values were obtained. All questions were asked in the Japanese language. The web-based survey was conducted over a 3-day period, from 12–14 October 2022.

All participants agreed to participate in the study and answered the survey questions. Participants’ personal information was protected by Macromill, Inc. [29]. The participants were given points that could be converted into cash. This study was approved by the Research Ethics Committee of Nippon Dental University College in Tokyo before the web-based survey was conducted (9 August 2022, approval No. 293).

### 2.2. Outcome Variable (WTP Values for Dental Checkups)

The outcome variable in this study was the WTP value for dental checkups. WTP values were obtained from the study participants based on the payment card method [12,30]. (The questionnaire is provided in a Supplementary File.) The participants were asked about the maximum amount they would be willing to pay to receive one dental checkup. As a proviso to this question, the following description was provided to the study participants: (1) “Under the Japanese medical insurance system, healthcare services for disease prevention are not covered by insurance. Please answer this question by assuming full payment at your own expense.” (2) “’Dental checkups’ in this survey refers to a checkup by a dentist to assess the condition of the teeth for the purpose of early detection of dental caries and periodontal disease (radiographs are obtained, if necessary). It does not include scaling of calculus or polishing of tooth surfaces.”

The study participants were presented with the following range of amounts for their responses: 0 yen, 1000 yen, 2000 yen, 3000 yen, 4000 yen, 5000 yen, 6000 yen, 7000 yen, 8000 yen, 9000 yen, 10,000 yen, 11,000 yen, 12,000 yen, 13,000 yen, 14,000 yen, 15,000 yen, 16,000 yen, 17,000 yen, 18,000 yen, 19,000 yen, and 20,000 yen or more (as of February 2023, 1000 yen = 7.5 USD). These ranges were set based on a previous study [22]. The questionnaire on WTP values for dental checkups was pretested before the actual survey was administered to the study participants.

Participants who responded “0 yen” were given additional questions to determine whether their reason was “true zeros” or “protest zeros” [14]. If the participant answered “The cost of dental checkups should be fully paid by the government, insurers, or other parties” as the reason for choosing 0 yen, this response was defined as a “protest zeros” response because it does not reflect an economic evaluation of healthcare services such as dental checkups [14]. Hence, these “protest zeros” responses were excluded when conducting the statistical analysis.

### 2.3. Explanatory Variables

The explanatory variables were set according to the individual characteristics of the study participants, which consisted of socioeconomic factors and oral health status. Socioeconomic factors included gender, age, household income, employment status, marital status, presence of children, and the municipality of residence. Variables related to oral health status included the number of teeth and frequency of tooth brushing.

The participants’ ages were categorized into the following five groups: 20–29 years, 30–39 years, 40–49 years, 50–59 years, and 60–69 years. Household income was categorized into six groups: <2 million yen, 2–4 million yen, 4–6 million yen, 6–8 million yen, ≥8 million yen, and unknown (As of 2020, the average household income of Japanese people was 5.64 million yen, and the median was 4.4 million yen [31].) Employment status was categorized into four groups: regular workers, homemakers, part-time workers, and not working and others. Marital status was categorized as married or single. The presence of children variable was categorized as having children or no children. The municipalities in which study participants resided were categorized into four groups based on the Japanese municipality system: metropolises (ordinance-designated cities with populations of ≥500,000 and the 23 wards of Tokyo), core cities (ordinance-designated cities with populations of ≥200,000), other cities (cities with populations of ≥50,000 excluding metropolises and core cities), and towns/villages (small municipalities that do not meet the specifications of cities).

The number of teeth in the study participants was categorized into three groups: <20, 20–27, and ≥28 teeth. The frequency of tooth brushing was categorized into four groups: ≥3 times daily, twice daily, once daily, and occasional/no brushing.

### 2.4. Statistical Analysis

First, descriptive statistics were calculated for each variable. The outcome variable (WTP values for dental checkups) was used as quantitative data, and the explanatory variables were used as categorical data. In addition, study participants were divided into two groups based on whether they received regular dental checkups (those who received regular dental checkups: RDC group; those who did not receive regular dental checkups: non-RDC group). The criterion for whether or not the participants received regular dental checkups was “whether or not they received dental checkups at least once a year,” based on a survey by the Ministry of Health, Labour, and Welfare [32].

Second, to understand the distribution of WTP values for dental checkups, graphs were created for the RDC and non-RDC groups. In addition, the descriptive statistics of the WTP values for both the RDC and non-RDC groups were calculated, and the two groups were compared after excluding the “protest zeros” responses, using the Mann-Whitney U test; this test was used because the WTP values did not follow the normal distribution.

Third, the association between the outcome variable (WTP values for dental checkups) and the explanatory variables (gender, age, household income, employment status, marital status, presence of children, municipality of residence, number of teeth, and frequency of tooth brushing) was evaluated using the Tobit regression model for the RDC and non-RDC groups. The Tobit regression model was used because the WTP values for dental checkups were either zero or more amounts but not negative amounts and because they exhibit characteristics as censored data [33]. In addition, Tobit regression was calculated using robust standard errors. With regard to the inclusion of explanatory variables, univariate and multivariate analyses were conducted after adjusting for all variables. In all analyses, the “protest zeros” responses were excluded.

In this study, Stata version 17 (StataCorp LLC, College Station, TX, USA) was used for statistical analysis. Statistical significance was set at *p* < 0.05.

## 3. Results

### 3.1. Demographic Characteristics of the Study Participants and the Number and Proportion of the RDC and Non-RDC Groups

Table 1 shows the demographic characteristics of the study participants (*n* = 3336) and the number and proportion of each when divided into the RDC group (*n* = 1785; 53.5%) and non-RDC group (*n* = 1551; 46.5%).

The Chi-squared test revealed statistically significant differences between the two groups in the following variables: gender, household income, employment status, marital status, municipalities, number of teeth, frequency of tooth brushing (*p* < 0.001), and presence of children (*p* = 0.004).

### 3.2. Distribution and Comparison of WTP Values for Dental Checkups between the RDC and Non-RDC Groups

**Figure 1:**
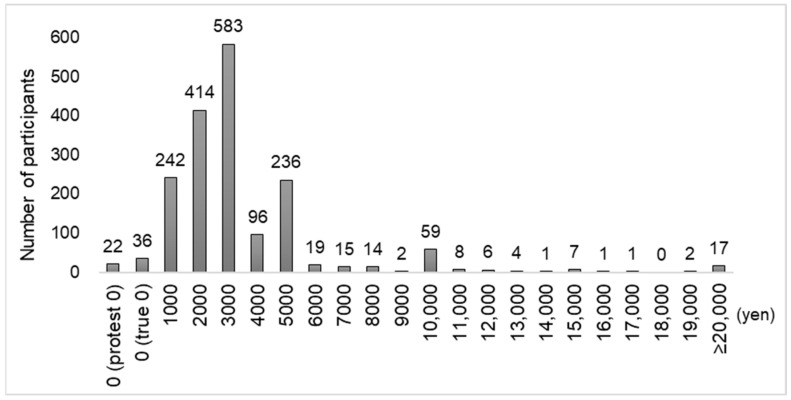
Distribution of WTP values for dental checkups in the RDC group.

**Figure 2:**
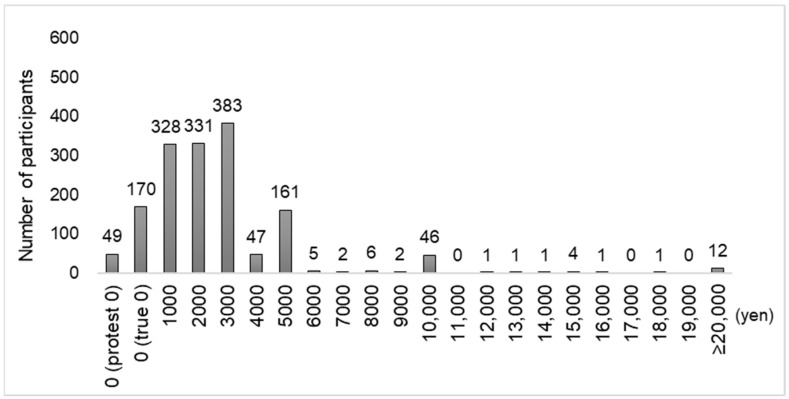
Distribution of WTP values for dental checkups in the non-RDC group.

The distribution of WTP values for dental checkups is shown in Figure 1 for the RDC group and Figure 2 for the non-RDC group. Table 2 shows the comparison of the descriptive statistics of WTP values for dental checkups in the RDC and non-RDC groups, excluding responses with protest zeros (RDC group: 22, non-RDC group: 49). In the RDC group (1763 participants), the median was 3000 yen (22.51 USD), the interquartile range was 2000–4000 yen (15.01–30.02 USD), and mean was 3439.6 yen (25.81 USD). In the non-RDC group (1502 participants), the median was 2000 yen (15.01 USD), the interquartile range was 1000–3000 yen (7.50–22.51 USD), and the mean was 2713.0 (20.36 USD) yen. The Mann-Whitney U test revealed a statistically significant difference between the RDC and non-RDC groups (*p* < 0.001).

### 3.3. Association between WTP Values for Dental Checkups and Study Participants’ Individual Characteristics in the RDC and Non-RDC Groups

The multivariate Tobit regression model demonstrated the association between WTP values for dental checkups and characteristics of the study participants for both the RDC and non-RDC groups (Table 3 and Table 4) (The results of the univariate Tobit analysis are shown in Supplementary Tables S1 and S2).

Regarding the WTP values for dental checkups in the RDC group (Table 3), age 50–59 years (coefficient: −515.14, 95%CI: −999.65 to −30.64), household income <2 million yen (coefficient: −543.95, 95%CI: −1081.14 to −6.77), homemaker and part-time worker employment status (homemaker, coefficient: −407.09, 95%CI: −780.41 to −33.78; part-time worker, coefficient: −408.48, 95%CI: −764.61 to −52.35), and having children (coefficient: −442.31, 95%CI: −779.65 to −104.98) were significantly associated with decreased WTP values, while male gender (coefficient: 329.48, 95%CI: 0.83 to 658.12), household incomes >8 million yen (coefficient: 600.55, 95%CI: 140.55 to 1060.56), and tooth brushing ≥3 times daily (coefficient: 473.18, 95%CI: 10.78 to 935.57) were associated with increased WTP values.

Regarding the WTP values for dental checkups in the non-RDC group (Table 4), age ≥30 years (30–39 years, coefficient: −741.24, 95%CI: −1354.07 to −128.41; 40–49 years, coefficient: −1200.37, 95%CI: −1762.87 to −637.88; 50–59 years, coefficient: −1034.32, 95%CI: −1591.12 to −477.53; 60–69 years, coefficient: −696.59, 95%CI: −1332.38 to −60.80), household incomes <4 million yen (<2 million yen, coefficient: −897.82, 95%CI: −1580.95 to −214.69; 2–4 million yen, coefficient: −529.94, 95%CI: −994.76 to −65.12), and presence of ≥28 teeth (coefficient: −362.48, 95%CI: −707.17 to −17.80) were significantly associated with decreased WTP values, while household incomes of ≥8 million (coefficient: 661.82, 95%CI: 110.49 to 1213.14) were associated with increased WTP values.

## 4. Discussion

### 4.1. Major Findings of This Study

Using a nationwide web-based survey, the WTP values for dental checkups in the RDC and non-RDC groups were ascertained and analyzed to assess their association with the study participants’ individual characteristics. As a result, two major points were revealed.

First, the median WTP value for dental checkups was 3000 yen (22.51 USD) (mean: 3439.6 yen [25.81 USD]) in the RDC group and 2000 yen (15.01 USD) (mean: 2713.0 yen [20.36 USD]) in the non-RDC group, which was a statistically significant difference between the two groups.

Second, age 50–59 years, lower household income, homemaker and part-time worker employment status, and having children were significantly associated with lower WTP values, while male gender, higher household income, and tooth brushing ≥3 times daily were associated with higher WTP values. In the non-RDC group (Table 4), age ≥30 years, lower household income, and presence of ≥28 teeth were significantly associated with lower WTP values, while higher household incomes were associated with higher WTP values.

Therefore, the results of this study suggest that the WTP values for dental checkups were lower in the non-RDC group than in the RDC group, and socioeconomic factors were associated with WTP values in both groups.

### 4.2. WTP Values for Dental Checkups in the RDC and Non-RDC Groups

Although many WTP studies have reported on dental treatment [15,16,17,18,19,20], few have focused on receiving dental checkups [21,22]. A previous study [22] related to the results of this study showed that the WTP values for dental checkups were evaluated by targeting patients in dental clinics, with a median WTP value of 2000 yen (mean: 2252.6 yen) for regular visitors and a median WTP value of 2000 yen (mean: 2124.9 yen) for infrequent visitors. The results of this study were different from the findings of the previous study; however, they are not simply comparable because the previous study surveyed patients visiting dental clinics, whereas, in this study, the sample was recruited from the general population that approximates the Japanese population, using the quota sampling method and conducting a web-based survey. Therefore, the results of this study are the first to determine WTP values for dental checkups in the general population nationwide and can be expected to contribute to health policy planning.

This study found that the RDC group responded with a higher value than the non-RDC group regarding the maximum amount they could pay for a dental checkup (RDC group: median 3000 yen, mean 3439.6 yen; non-RDC group: median 2000 yen, mean 2713.0 yen). Several previous studies have suggested that those who habitually receive regular dental checkups have an increased awareness of oral health [34,35]. Therefore, the results of this study may also have been influenced by the fact that the RDC group gave more importance to receiving dental checkups to maintain their oral health than the non-RDC group. Another possible factor affecting the WTP value is its association with household income, as described in Section 4.3.

### 4.3. Association between WTP Values for Dental Checkups and Individual Characteristics in the RDC and Non-RDC Groups

There was a positive correlation between WTP values and household income in both the RDC and non-RDC groups. Those with lower household incomes were likelier to report lower WTP values for dental checkups. Of particular note is that in the non-RDC group, associations were observed in a wide range of age groups over 30 years. In addition, the non-RDC group responded with significantly lower WTP values for dental checkups than the RDC group. Several previous studies have shown that income limitations are a barrier to regular dental attendance [8,9,10,11], and the results of this study support these previous findings from the perspective of economic evaluation of dental checkups. Based on these findings, it can be implied that compared to the RDC group, there is a limitation to the maximum amount that can be paid for dental checkups within a wide age range in the non-RDC group and that this may be associated with economic background factors; hence, this suggests the need for policy interventions.

Further, homemakers, part-time workers, and those with children responded with lower WTP values only in the RDC group. Even if these participants were in the habit of receiving regular dental checkups, they might be limited in the amount of cost they can spend on dental checkups; therefore, it is possible that they reported low WTP values. Moreover, the results of this study showed that men had higher WTP values for dental checkups than women. Previous studies have shown that women are more likely to have an increased awareness of oral health than men [11,36]. However, according to a report by Japan’s Ministry of Health, Labour and Welfare, the income of working men is about 2.1 times that of working women [31], and the reason for this is reportedly due to the differences in employment positions and length of service between men and women [37]. Therefore, it is possible that men in the RDC group answered that they could afford to spend on dental checkups because of their financial stability rather than because of their awareness of oral health.

Regarding oral health conditions, in the RDC group, those who brushed their teeth ≥3 times daily reported higher WTP values. This result suggests that participants in the RDC group had a high awareness of oral health [34,35] and identified the importance of dental checkups. In contrast, in the non-RDC group, those with ≥28 teeth had lower WTP values. The reason for this may be that they have a full set of teeth and have no trouble chewing; therefore, they have little awareness of the need to protect their oral health. However, this causal relationship remains unclear and requires further investigation.

Regarding the municipality in which the study participants resided, there was no statistically significant association in either the RDC or non-RDC group. Generally, there are reportedly more barriers in rural areas than in urban areas with regard to access to dental services [10,38]. However, there is reportedly little inequality in the geographic distribution of the number of dental clinics in Japan [39]. That is, there are fewer differences in barriers to access to dental services between rural and urban areas; as a result, there may have been less impact on WTP values for both groups in this study.

### 4.4. Implications for Health Policy of This Study

Under the Japanese medical insurance system, most dental treatments, such as caries treatment, endodontic treatment, periodontal disease treatment, and prosthetic treatment, are covered by insurance. However, preventive practices such as dental checkups are not covered by insurance [23]. The results of this study showed that in both the RDC and non-RDC groups, those with a lower household income were more likely to report a lower maximum amount they could pay for dental checkups. This result raises concerns, particularly among the non-RDC group, who may face barriers to accessing dental services due to economic reasons.

Universal Health Coverage (UHC) is one of the Sustainable Development Goals (SDGs) advocated by the United Nations in 2015 [40], and the Japanese medical insurance system may be considered to be achieving UHC. However, several Japanese studies have suggested that income limitations may affect access to dental services [8,11]. Therefore, the establishment of a system that allows people to receive dental checkups without co-payment, using public funds and other financial resources, may result in improved access to dental services [41,42]. It is necessary for policymakers to plan health policies that consider socioeconomic factors, such as people’s incomes, to ensure equality of oral health status.

### 4.5. Limitations of This Study

This study has several limitations. First, in WTP studies, there are several methods to obtain WTP values from study participants, and each method has its advantages and disadvantages [12,14]. This study used a payment card as the appropriate method because a web-based survey was used to obtain the WTP values for dental checkups from study participants on a nationwide scale. Therefore, this method may have had range bias since the study participants’ choices were bound by the amount in the payment card they were presented with [30]. Second, although the study sample approximated the Japanese population using a quota sampling method, sampling bias cannot be completely ruled out because the study participants were selected from among those registered with a web-based survey company. Internet usage among the Japanese is increasing [43]; however, the possibility of sampling bias remains a concern in web-based surveys [44]. Third, this was a cross-sectional study conducted using a web-based survey for the study participants. Although this study revealed the individual characteristics of the study participants associated with high/low WTP values in both the RDC and non-RDC groups, it was not possible to determine a causal relationship between their factors due to the cross-sectional design of the study.

## 5. Conclusions

Based on the results of this study, the null hypothesis stated in the objective was rejected, and the following conclusions were obtained: (1) the WTP values for dental checkups were lower in the non-RDC group than in the RDC group, and (2) there was a significant association between high/low WTP values and socioeconomic factors in both groups; in particular, in the non-RDC group, those with a lower household income aged ≥30 years were more likely to propose lower WTP values for dental checkups.

Hence, this result suggests the need for policy intervention to improve access to regular dental checkups.

## Figures and Tables

**Table 1:** Demographic characteristics of the study participants and the number and proportion of the RDC and non-RDC groups.

	Total		Whether or Not the Participants Receive RDC				
			RDC Group		Non-RDC Group		*p*-Value *
Total	3336	(100.0)	1785	(53.5)	1551	(46.5)	
Gender, n (%)							
Men	1678	(50.3)	785	(44.0)	893	(57.6)	<0.001
Women	1658	(49.7)	1000	(56.0)	658	(42.4)	
Age, n (%)							
20–29 years	524	(15.7)	279	(15.6)	245	(15.8)	0.300
30–39 years	609	(18.3)	311	(17.4)	298	(19.2)	
40–49 years	794	(23.8)	417	(23.4)	377	(24.3)	
50–59 years	723	(21.7)	388	(21.7)	335	(21.6)	
60–69 years	686	(20.6)	390	(21.9)	296	(19.1)	
Household income, n (%)							
<2 million yen	328	(9.8)	165	(9.2)	163	(10.5)	<0.001
2–4 million yen	577	(17.3)	298	(16.7)	279	(18.0)	
4–6 million yen	630	(18.9)	347	(19.4)	283	(18.3)	
6–8 million yen	483	(14.5)	275	(15.4)	208	(13.4)	
≥8 million yen	601	(18.0)	361	(20.2)	240	(15.5)	
Unknown	717	(21.5)	339	(19.0)	378	(24.4)	
Employment status, n (%)							
Regular worker	1799	(53.9)	976	(54.7)	823	(53.1)	<0.001
Homemaker	537	(16.1)	307	(17.2)	230	(14.8)	
Part-time worker	499	(15.0)	283	(15.9)	216	(13.9)	
Not working and others	501	(15.0)	219	(12.3)	282	(18.2)	
Marital status, n (%)							
Married	1959	(58.7)	1109	(62.1)	850	(54.8)	<0.001
Single	1377	(41.3)	676	(37.9)	701	(45.2)	
Presence of children, n (%)							
Having children	1742	(52.2)	973	(54.5)	769	(49.6)	0.004
No children	1594	(47.8)	812	(45.5)	782	(50.4)	
Municipalities, n (%)							
Metropolis (pop 500,000+)	1162	(34.8)	667	(37.4)	495	(31.9)	<0.001
Core cities (pop 200,000+)	573	(17.2)	308	(17.3)	265	(17.1)	
Cities (pop 50,000+)	1253	(37.6)	656	(36.8)	597	(38.5)	
Towns/villages	348	(10.4)	154	(8.6)	194	(12.5)	
Number of teeth, n (%)							
≥28	1863	(55.9)	936	(52.4)	927	(59.8)	<0.001
20–27	1196	(35.9)	704	(39.4)	492	(31.7)	
<20	277	(8.3)	145	(8.1)	132	(8.5)	
Frequency of brushing teeth, n (%)							
≥ three times daily	958	(28.7)	588	(32.9)	370	(23.9)	<0.001
Twice daily	1684	(50.5)	908	(50.9)	776	(50.0)	
Once daily	608	(18.2)	267	(15.0)	341	(22.0)	
Occasional/no brushing	86	(2.6)	22	(1.2)	64	(4.1)	

Note: RDC = regular dental checkups; RDC group = group of participants who received regular dental checkups; non-RDC group = group of participants who did not receive regular dental checkups; * Chi-square test.

**Table 2:** Comparison of WTP values for dental checkups in the RDC group and the non-RDC group.

	Number	Median	Interquartile Range	Mean	SD	*p*-Value *
RDC group	1763	3000	2000–4000	3439.6	2863.4	<0.001
Non-RDC group	1502	2000	1000–3000	2713.0	2711.2	

Note: RDC = regular dental checkups; RDC group = group of participants who received regular dental checkups; non-RDC group = group of participants who did not receive regular dental checkups; Calculated excluding answers with protest zeros; * Mann-Whitney U test.

**Table 3:** Association between WTP values for dental checkups and study participants’ characteristics in the RDC group (multivariate Tobit regression model).

	Coefficient	Robust Standard Error	t	*p*-Value	95% Confidence Interval	
Gender						
Men	329.48	167.56	1.97	0.049	0.83	658.12
Women	Reference					
Age						
20–29 years	Reference					
30–39 years	−131.08	259.46	−0.51	0.613	−639.97	377.80
40–49 years	−448.91	236.46	−1.90	0.058	−912.68	14.87
50–59 years	−515.14	247.03	−2.09	0.037	−999.65	−30.64
60–69 years	−248.10	274.70	−0.90	0.367	−786.87	290.68
Household income						
<2 million yen	−543.95	273.89	−1.99	0.047	−1081.14	−6.77
2–4 million yen	−368.10	213.22	−1.73	0.084	−786.31	50.10
4–6 million yen	Reference					
6–8 million yen	413.15	242.74	1.70	0.089	−62.94	889.24
≥8 million yen	600.55	234.54	2.56	0.011	140.55	1060.56
Unknown	−600.99	206.68	−2.91	0.004	−1006.37	−195.62
Employment status						
Regular worker	Reference					
Homemaker	−407.09	190.34	−2.14	0.033	−780.41	−33.78
Part-time worker	−408.48	181.57	−2.25	0.025	−764.61	−52.35
Not working and others	−332.33	263.18	−1.26	0.207	−848.51	183.84
Marital status						
Married	−11.67	211.27	−0.06	0.956	−426.04	402.69
Single	Reference					
Presence of children						
Having children	−442.31	171.99	−2.57	0.010	−779.65	−104.98
No children	Reference					
Municipalities						
Metropolis (pop 500,000+)	261.49	154.65	1.69	0.091	−41.82	564.81
Core cities (pop 200,000+)	320.56	198.19	1.62	0.106	−68.15	709.27
Cities (pop 50,000+)	Reference					
Towns/villages	102.42	211.59	0.48	0.628	−312.58	517.42
Number of teeth						
≥28	−198.89	148.28	−1.34	0.180	−489.71	91.93
20–27	Reference					
<20	520.03	327.96	1.59	0.113	−123.21	1163.26
Frequency of brushing teeth						
≥ three times daily	473.18	235.75	2.01	0.045	10.78	935.57
Twice daily	102.26	215.90	0.47	0.636	−321.18	525.71
Once daily	Reference					
Occasional/no brushing	−1050.26	724.83	−1.45	0.148	−2471.90	371.38
Constant	3727.30	365.07	10.21	<0.001	3011.29	4443.32

Note: RDC = regular dental checkups; RDC group = group of participants who received regular dental checkups; WTP = willingness to pay; Calculated by excluding answers with protest zeros; number of observations = 1763, log pseudolikelihood = −16,205.10, *p* < 0.001.

**Table 4:** Association between WTP values for dental checkups and study participants’ characteristics in the non-RDC group (multivariate Tobit regression model).

	Coefficient	Robust Standard Error	t	*p*-Value	95% Confidence Interval	
Gender						
Men	−144.09	189.81	−0.76	0.448	−516.42	228.25
Women	Reference					
Age						
20–29 years	Reference					
30–39 years	−741.24	312.42	−2.37	0.018	−1354.07	−128.41
40–49 years	−1200.37	286.76	−4.19	<0.001	−1762.87	−637.88
50–59 years	−1034.32	283.85	−3.64	<0.001	−1591.12	−477.53
60–69 years	−696.59	324.12	−2.15	0.032	−1332.38	−60.80
Household income						
<2 million yen	−897.82	348.26	−2.58	0.010	−1580.95	−214.69
2–4 million yen	−529.94	236.96	−2.24	0.025	−994.76	−65.12
4–6 million yen	Reference					
6–8 million yen	5.57	258.70	0.02	0.983	−501.88	513.02
≥8 million yen	661.82	281.06	2.35	0.019	110.49	1213.14
Unknown	−796.94	236.62	−3.37	0.001	−1261.09	−332.78
Employment status						
Regular worker	Reference					
Homemaker	96.39	257.36	0.37	0.708	−408.43	601.21
Part-time worker	−188.17	214.69	−0.88	0.381	−609.29	232.96
Not working and others	−45.01	253.68	−0.18	0.859	−542.62	452.60
Marital status						
Married	−99.27	227.71	−0.44	0.663	−545.94	347.39
Single	Reference					
Presence of children						
Having children	−240.52	213.28	−1.13	0.260	−658.89	177.84
No children	Reference					
Municipalities						
Metropolis (pop 500,000+)	101.54	187.46	0.54	0.588	−266.17	469.26
Core cities (pop 200,000+)	−153.81	187.99	−0.82	0.413	−522.57	214.95
Cities (pop 50,000+)	Reference					
Towns/villages	17.33	274.95	0.06	0.950	−522.01	556.67
Number of teeth						
≥28	−362.48	175.72	−2.06	0.039	−707.17	−17.80
20–27	Reference					
<20	256.99	392.33	0.66	0.513	−512.60	1026.57
Frequency of brushing teeth						
≥ three times daily	−72.96	225.33	−0.32	0.746	−514.96	369.04
Twice daily	−184.76	202.18	−0.91	0.361	−581.35	211.83
Once daily	Reference					
Occasional/no brushing	782.39	789.20	0.99	0.322	−765.68	2330.46
Constant	4143.66	446.54	9.28	<0.001	3267.74	5019.58

Note: RDC = regular dental checkups; WTP = willingness to pay; non-RDC group = group of participants who did not receive regular dental checkups; Calculated by excluding answers with protest zeros; number of observations = 1502, log pseudolikelihood = −12,679.89, *p* < 0.001.

## Data Availability

Data could not be shared publicly because the participants provided no informed consent for open data sharing.

## Supplementary Materials

The following supporting information can be downloaded at: https://www.mdpi.com/article/10.3390/ijerph20054145/s1. Supplementary File S1: Questionnaire provided to the study participants; Table S1: Association between WTP values for dental checkups and study participants’ characteristics in the RDC group (univariate tobit regression analysis); Table S2: Association between WTP values for dental checkups and study participants’ characteristics in the non-RDC group (univariate tobit regression analysis; Supplementary File S2: STROBE Statement—Checklist of items that should be included in reports of cross-sectional studies.
