# Supplementary material for: People’s Willingness to Pay for Dental Checkups and the Associated Individual Characteristics: A Nationwide Web-Based Survey among Japanese Adults
Source: Int J Environ Res Public Health. 2023 Feb 25;20(5):4145. doi: 10.3390/ijerph20054145 (PMC10001831; doi:10.3390/ijerph20054145)
Supplement: Supplementary file 1 [file ijerph-20-04145-s001.zip › ijerph-2226307-supplementary.pdf]

Supplementary file

■Questionnaire provided to the study participants
(In the actual study, the participants answered this questionnaire in the Japanese language on the web.)

What is the maximum amount you would be willing to pay to receive one dental checkup? (Select only one)

Note:
- Under the Japanese medical insurance system, healthcare services for disease prevention are not covered by insurance. Please answer this question by assuming full payment at your own expense.
- “Dental checkups” in this survey refers to a checkup by a dentist to assess the condition of the teeth for the purpose of early detection of dental caries and periodontal disease (radiographs are obtained, if necessary). It does not include scaling of calculus or polishing of tooth surfaces.

□ 0 yen	□ 7000 yen	□ 14000 yen
□ 1000 yen	□ 8000 yen	□ 15000 yen
□ 2000 yen	□ 9000 yen	□ 16000 yen
□ 3000 yen	□ 10000 yen	□ 17000 yen
□ 4000 yen	□ 11000 yen	□ 18000 yen
□ 5000 yen	□ 12000 yen	□ 19000 yen
□ 6000 yen	□ 13000 yen	□ 20000 yen or more

■Additional question
(Only participants who answered “0 yen” were asked further questions.)

What is the reason for choosing “0 yen”? (Select only one)

□ I cannot afford to pay financially.
□ I do not have time to receive dental checkups.
□ I do not think it is necessary to receive dental checkups.
□ The cost of dental checkups should be fully paid by the government, insurers, or other parties.
□ Others

Supplementary Table S1

Table S1 Association between WTP values for dental checkups and study participants' characteristics in the RDC group (univariate tobit regression analysis)

	Coefficient	Robust standard error	t	p-Value	95% confidence interval	
Gender						
Men	672.08	144.75	4.64	<0.001	388.18	955.98
Women	Reference					
Age						
20–29 years	Reference					
30–39 years	-156.75	259.25	-0.60	0.546	-665.22	351.73
40–49 years	-429.23	230.14	-1.87	0.062	-880.60	22.13
50–59 years	-586.06	236.13	-2.48	0.013	-1049.19	-122.93
60–69 years	-460.95	239.80	-1.92	0.055	-931.28	9.37
Household income						
<2 million yen	-695.77	262.00	-2.66	0.008	-1209.64	-181.91
2–4 million yen	-368.57	215.57	-1.71	0.087	-791.37	54.23
4–6 million yen	Reference					
6–8 million yen	379.08	247.02	1.53	0.125	-105.39	863.56
≥ 8 million yen	641.52	237.75	2.70	0.007	175.23	1107.82
Unknown	-709.09	205.18	-3.46	0.001	-1111.51	-306.68
Employment status						
Regular worker	Reference					
Homemaker	-907.83	142.67	-6.36	<0.001	-1187.66	-628.01
Part-time worker	-928.90	163.13	-5.69	<0.001	-1248.86	-608.95
Not working and others	-655.38	257.86	-2.54	0.011	-1161.12	-149.65
Marital status						
Married	-140.45	148.90	-0.94	0.346	-432.50	151.59
Single	Reference					
Presence of children						
Having children	-435.78	142.16	-3.07	0.002	-714.59	-156.97
No children	Reference					
Municipalities						
Metropolis (pop 500,000+)	453.32	160.26	2.83	0.005	139.00	767.64
Core cities (pop 200,000+)	505.30	205.78	2.46	0.014	101.71	908.89
Cities (pop 50,000+)	Reference					
Towns/villages	39.50	217.34	0.18	0.856	-386.78	465.78
Number of teeth						
≥ 28	-105.22	143.10	-0.74	0.462	-385.89	175.45
20–27	Reference					
<20	591.55	350.04	1.69	0.091	-94.98	1278.08
Frequency of brushing teeth						
≥ three times daily	354.57	243.68	1.46	0.146	-123.37	832.51
Twice daily	54.74	225.05	0.24	0.808	-386.66	496.14
Once daily	Reference					
Occasional/no brushing	-873.79	788.22	-1.11	0.268	-2419.73	672.15

Note: RDC = regular dental checkups; RDC group = group of participants who received regular dental checkups; WTP = willingness to pay; Calculated by excluding answers with protest zeros; number of observations = 1763

Supplementary Table S2

Table S2 Association between WTP values for dental checkups and study participants' characteristics in the non-RDC group (univariate tobit regression analysis)

	Coefficient	Robust standard error	t	p-Value	95% confidence interval	
Gender						
Men	152.39	152.91	1.00	0.319	-147.56	452.33
Women	Reference					
Age						
20–29 years	Reference					
30–39 years	-649.34	311.52	-2.08	0.037	-1260.40	-38.28
40–49 years	-1044.92	280.48	-3.73	<0.001	-1595.10	-494.73
50–59 years	-832.83	278.06	-3.00	0.003	-1378.26	-287.41
60–69 years	-525.73	301.79	-1.74	0.082	-1117.71	66.24
Household income						
<2 million yen	-659.58	325.52	-2.03	0.043	-1298.11	-21.05
2–4 million yen	-397.31	223.03	-1.78	0.075	-834.79	40.17
4–6 million yen	Reference					
6–8 million yen	-207.39	259.07	-0.80	0.424	-715.58	300.79
≥ 8 million yen	500.53	277.19	1.81	0.071	-43.19	1044.25
Unknown	-728.65	236.18	-3.09	0.002	-1191.93	-265.37
Employment status						
Regular worker	Reference					
Homemaker	-164.56	210.18	-0.78	0.434	-576.84	247.72
Part-time worker	-452.47	192.24	-2.35	0.019	-829.54	-75.39
Not working and others	-134.46	261.26	-0.51	0.607	-646.94	378.02
Marital status						
Married	-64.02	159.96	-0.40	0.689	-377.79	249.76
Single	Reference					
Presence of children						
Having children	-207.90	153.62	-1.35	0.176	-509.23	93.43
No children	Reference					
Municipalities						
Metropolis (pop 500,000+)	229.06	190.67	1.20	0.230	-144.95	603.07
Core cities (pop 200,000+)	-112.95	191.37	-0.59	0.555	-488.33	262.42
Cities (pop 50,000+)	Reference					
Towns/villages	39.50	284.85	0.14	0.890	-519.24	598.24
Number of teeth						
≥ 28	-225.41	160.24	-1.41	0.160	-539.73	88.90
20–27	Reference					
<20	389.10	448.45	0.87	0.386	-490.55	1268.75
Frequency of brushing teeth						
≥ three times daily	-50.92	226.40	-0.22	0.822	-495.01	393.17
Twice daily	-84.09	201.76	-0.42	0.677	-479.85	311.67
Once daily	Reference					
Occasional/no brushing	890.45	877.62	1.01	0.310	-831.04	2611.95

Note: Note: RDC = regular dental checkups; WTP = willingness to pay; non-RDC group = group of participants who did not receive regular dental checkups; Calculated by excluding answers with protest zeros; number of observations = 1502

Supplementary file: STROBE Statement

STROBE Statement—Checklist of items that should be included in reports of ***cross-sectional studies***

	Item No	Recommendation	Page No
**Title and abstract**	1	(*a*) Indicate the study's design with a commonly used term in the title or the abstract	1
		(*b*) Provide in the abstract an informative and balanced summary of what was done and what was found	1
**Introduction**			
Background/rationale	2	Explain the scientific background and rationale for the investigation being reported	1-2
Objectives	3	State specific objectives, including any prespecified hypotheses	2
**Methods**			
Study design	4	Present key elements of study design early in the paper	2
Setting	5	Describe the setting, locations, and relevant dates, including periods of recruitment, exposure, follow-up, and data collection	2-3
Participants	6	(*a*) Give the eligibility criteria, and the sources and methods of selection of participants	2-3
Variables	7	Clearly define all outcomes, exposures, predictors, potential confounders, and effect modifiers. Give diagnostic criteria, if applicable	3-4
Data sources/ measurement	8*	For each variable of interest, give sources of data and details of methods of assessment (measurement). Describe comparability of assessment methods if there is more than one group	3-4
Bias	9	Describe any efforts to address potential sources of bias	2-3
Study size	10	Explain how the study size was arrived at	2-3
Quantitative variables	11	Explain how quantitative variables were handled in the analyses. If applicable, describe which groupings were chosen and why	3-4
Statistical methods	12	(*a*) Describe all statistical methods, including those used to control for confounding	4
		(*b*) Describe any methods used to examine subgroups and interactions	N/A
		(*c*) Explain how missing data were addressed	3
		(*d*) If applicable, describe analytical methods taking account of sampling strategy	N/A
		(*e*) Describe any sensitivity analyses	N/A
**Results**			
Participants	13*	(a) Report numbers of individuals at each stage of study—eg numbers potentially eligible, examined for eligibility, confirmed eligible, included in the study, completing follow-up, and analysed	4-9
		(b) Give reasons for non-participation at each stage	6-9
		(c) Consider use of a flow diagram	N/A
Descriptive data	14*	(a) Give characteristics of study participants (eg demographic, clinical, social) and information on exposures and potential confounders	4-5 Table1
		(b) Indicate number of participants with missing data for each variable of interest	N/A
Outcome data	15*	Report numbers of outcome events or summary measures	6 Figure1,2 Table2
Main results	16	(*a*) Give unadjusted estimates and, if applicable, confounder-adjusted estimates and their precision (eg, 95% confidence interval). Make clear which confounders were adjusted for and why they were included	7-9 Table3,4 S1,S2
		(*b*) Report category boundaries when continuous variables were categorized	N/A
		(*c*) If relevant, consider translating estimates of relative risk into absolute risk for a meaningful time period	N/A
Other analyses	17	Report other analyses done—eg analyses of subgroups and interactions, and sensitivity analyses	N/A

**Discussion**			
Key results	18	Summarise key results with reference to study objectives	9
Limitations	19	Discuss limitations of the study, taking into account sources of potential bias or imprecision. Discuss both direction and magnitude of any potential bias	11
Interpretation	20	Give a cautious overall interpretation of results considering objectives, limitations, multiplicity of analyses, results from similar studies, and other relevant evidence	9-11
Generalisability	21	Discuss the generalisability (external validity) of the study results	10-11
**Other information**			
Funding	22	Give the source of funding and the role of the funders for the present study and, if applicable, for the original study on which the present article is based	11

*Give information separately for exposed and unexposed groups.

Note: An Explanation and Elaboration article discusses each checklist item and gives methodological background and published examples of transparent reporting. The STROBE checklist is best used in conjunction with this article (freely available on the Web sites of PLoS Medicine at http://www.plosmedicine.org/, Annals of Internal Medicine at http://www.annals.org/, and Epidemiology at http://www.epidem.com/). Information on the STROBE Initiative is available at www.strobe-statement.org.
